# Knowledge and Practices of Pregnant Women regarding Oral Health in a Tertiary Care Hospital in Nepal

**DOI:** 10.31729/jnma.4420

**Published:** 2019-06-30

**Authors:** Neha Gupta, Manisha Chhetry

**Affiliations:** 1 Department of Dental surgery, Nobel Medical College and Teaching Hospital, Biratnagar, Nepal; 2 Department of Obstetrics and Gynecology, BP Koirala Institute of Health Sciences, Dharan, Nepal

**Keywords:** *antenatal care*, *oral health*, *perinatal*, *pregnancy*

## Abstract

**Introduction:**

Poor dental hygiene has been associated with various perinatal complications in studies done worldwide but few studies in Nepal have explored the knowledge of pregnant ladies regarding dental hygiene. The aim of the study was to know the knowledge and practices of pregnant women regarding oral health in a tertiary care center in Nepal.

**Methods:**

A qualitative study was carried out in Nobel Medical College and Teaching Hospital from January 15, 2018 to June 15, 2018 after approval from the Institutional Review Committee of Nobel Medical College. Convenience sampling was done. Fifty pregnant women admitted in antenatal ward were interviewed regarding their knowledge of dental care in pregnancy, the common dental problems they faced and the treatment taken. A predesigned proforma was used and results were analyzed using SPSS version 17.

**Results:**

Twenty two (44%) patients reported dental problems during pregnancy. Bleeding gums was seen in 7 (14%) and toothache in 7 (14%) were commonly reported dental problems. Forty seven (94%) patients acknowledged that routine dental care was needed for health, only 6 (12%) were aware that poor dental health could affect baby weight. Oral health not seen as priority in 24 (48%) was the main barrier to seeking dental care in pregnancy followed by costs of treatment in 18 (36%) and safety concerns in pregnancy in 8 (16%) cases.

**Conclusions:**

Though dental problems were a common occurrence in pregnancy, utilization of services was low for the same. The participants reported significant barriers to obtaining dental care including lack of knowledge about the importance of maternal oral health and the treatment costs.

## INTRODUCTION

Oral hygiene and its adverse perinatal outcome is an often neglected issue especially in a developing country like Nepal. With growing body of evidence, maintaining oral health in pregnancy has been recognized as an important health concern worldwide and neglect has been proven to be associated with adverse pregnancy outcomes like low birth weight, premature pre-labor rupture of membrane and prematurity with all its complications. The safety of dental treatment during pregnancy and its impact on improved perinatal outcomes have been well established in recent years.^13^

However, pregnant women often don't seek dental advice and treatment during pregnancy.^4^ Therefore to address this issue, it is fundamental to know the knowledge and prevalent practices regarding oral hygiene among the pregnant ladies.^5^

The aim of the study is to know the knowledge of pregnant women about oral health, to know the common dental problems in pregnancy and to identify the barriers in seeking care for dental problems in pregnancy in a tertiary care hospital of Nepal.

## METHODS

This is a qualitative study where a survey of pregnant women admitted in antenatal ward in Nobel Medical College and Teaching Hospital in Nepal was undertaken from January 15, 2018 to June 15, 2018. Ethical approval was obtained from the Institutional Review Committee of Nobel Medical College and written informed consent was obtained from all participants. All pregnant ladies who were admitted to the maternity ward due to various complications of pregnancy or for delivery irrespective of the period of gestation and those who were willing to be included in the study were enrolled. Patients who did not give consent or were unable to give interview were excluded.

Convenience sampling method was used. Data was collected from 50 pregnant women and the participants were interviewed as per the pre-designed proforma. All data collected were entered in Excel and analyzed using SPSS version 17.

## RESULTS

The most common oral health problem reported by participants were bleeding gums in 7 (14%) followed by toothache in 7 (14%) participants (Table 1).

**Table 1.** Dental problems reported by the patients.

Patient reported problems	n (%)
None	28 (56)
Bleeding gum	7 (14)
Sensitivity	4 (8)
Toothache	7 (14)
Cavities	3 (6)
Loose tooth	0 (0)
Others	1 (2)

**Figure 1.**
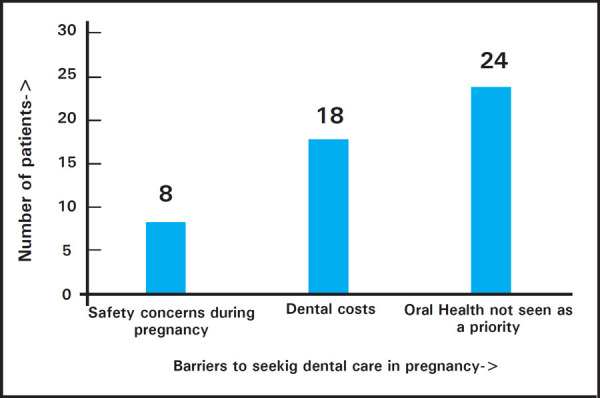
Barriers to seeking dental care in pregnancy.

The main barriers to seeking dental care for these women were oral health not seen as a priority in 24 (48%), dental costs in 18 (36%) and safety concerns regarding dental treatment during pregnancy in 8 (16%) participants (Figure 1).

Further, only 5 (10%) of pregnant women had seen a dentist in between 6 to 12 months. In terms of oral hygiene habits only 18 (36%) of women reported brushing twice daily which indicate inadequate knowledge about maternal and infant oral health, especially relating to good oral hygiene habits during the prenatal period (Table 2).

**Table 2.** Dental care of pregnant women.

Variables		n (%)
How often do you brush?		
	once a day	32 (64)
	Twice a day	18 (36)
Product used		
	Flouride toothpaste	45 (90)
Traditional products (eg.		5 (10)
Neem sticks” datwan”)		
When was the last time you saw a dentist?		
	<6months	1 (2)
	6 to<12months	4 (8)
	1yr to<2yrs	3 (6)
	2yrs to<5	8 (16)
	>5yrs	34 (68)

However, analysis of individual knowledge items showed that pregnant women had inadequate knowledge about potential impact of poor maternal oral health. Only 7 (14%) women were aware that dental decay could spread from the mother to the baby's mouth and only 6 (12%) women knew that mother's poor oral health may contribute to low birth weight baby. It is also apparent that some confusion exists among pregnant women regarding acquiring dental care both during pregnancy and early childhood. Nearly 42 (84%) women were unsure about the best time for first dental visit for a baby (Table 3).

**Table 3.** Percentage of correct participant responses by individual survey items.

Item content	Correct response (%)
Floss should be done daily to clean in between teeth	32%
Routine dental visit help keep teeth and gums healthy	94%
Dental decay can spread from the motherto the babys mouth	14%
A mothers poor oral health may contribute to low birth weight baby	12%
The first tooth usually appears at around 6 months of age	70%
Sleeping with bottle containing formula could cause caries in baby	72%
First dental visit should be done between 2-3 yrs.	12%

The age of expectant mothers were in the range of 17-38 years, with mean of 25.12±5.4. Twenty eight (56%) were primipara and 28 (56%) of women had finished their primary education (Table 4). Forty five (90%) women were in their third trimester.

**Table 4.** Socio demographic characteristics of patients.

Demographic variable	n (%)
<20yrs	6 (12)
20-30yrs	34 (68)
≥30yrs	10 (20)
<37wks	1 (2)
37-40 wks	4 (8)
>40wks	45 (90)
Illiterate	4 (8)
Primary	28 (56)
Secondary and higher	16 (32)
Parity	
Primi	28 (56)
Multi	22 (44)

## DISCUSSION

In this survey, pregnant mothers had poor knowledge regarding dental health during pregnancy. Mean age of the pregnant women in our study was of 25.12±5.4 years and 56% of them were in their first pregnancy while in the study done by Yolanda Martinez- Beneytoa et al^6^ mean age was 30 years and 57.7% were expecting their first child. Similar results were observed by Adeniyi et al.^7.^This may be because of early marriage and early childbearing in our country and lower level of literacy. Ninety percent women surveyed revealed they did not attend the dentist during previous twelve months. These results were poorer as compared to study conducted in Sydney where 69.5% of pregnant women did not receive dental care during their most recent pregnancy.^8^ Similar studies done by Hullah et al showed that 33% of the English women visited the dentist during pregnancy.^9,10^

In a study done in Jaipur, India by Mital et al the authors found that 42.5% of pregnant women brushed their teeth twice a day whereas in our study it was only 36%.^11^ Similar studies done in Denmark showed a 96% of subjects brushed their teeth twice a day. ^12^ In this study most of the pregnant women used flouride toothpaste (90%) whereas 10% used traditional products (eg. Neem sticks-datwan). Similar studies done in Sydney showed more than two thirdof pregnant women brush twice a day using flouridated toothpaste (98.3%), mouthwash (40.7%) and dental floss (42.7%) or other oral hygiene products used.^8^ In our set up knowledge and use regarding dental products other than toothpaste was very poor. Only 10% of the subjects seeked dental consultation in last one year which is similar to study done in our neighbouring country India by Mittal et al^11^ (11%) whereas utilization rates were higher (45.6%) in study done by George et al.^8^

One of main reason for poor maternal oral health is the physiological changes in hormone levels and dietary changes during this period which predispose the pregnant women to various dental problems.^10^ This study showed that 44% of patients had reported dental problem which was comparable to studies done by George et al^8^ where it was around 46%. But only 10% of these patients had visit to dentist in last one year even when a problem existed. The low use of dental services among pregnant women is well documented worldwide more so in underdeveloped and developing countries like Nepal.^4,13–15^ The low use of dental services in our study was mainly due to (48%) oral health not seen as priority followed by high cost of dental services (36%) and safety concern (16%). Similar study done by George et al. showed cost of dental services as main barrier for seeking dentist (29.2%).^8^ This highlights the basic lack of knowledge regarding the ill effects of dental problems on pregnancy. It is well documented that high dental cost are significant barrier for pregnant women seeking dental care worldwide.^2,16^

The result from this study was almost same with study done in Sydney^8^ about 97.9% of pregnant women agreed that routine dental visit help keep teeth and gums healthy. Only 12% of women acknowledged that poor oral health may contribute to low birth weight baby and first dental visit should be done between 2-3 yrs which was poorer as compared to other studies in developed countries.^7,8^ In this study reported 72% of women showed knowledge that, sleeping with bottle containing formula could cause caries in babywhereas study done by George et al^3^ shown slightly higher rate (84%)of women gave correct response. We can say that the dental knowledge and oral health practices are much better in developed countries when compared with that in Nepal. In this study most commonly reported dental problems during pregnancy were bleeding gums (14%) and toothache (14%), cavities (6%) and sensitivity (8%) which was similar to studies done elsewhere.^7,8^

The study has been done in small settings so the results cannot be generalized. It is recommended that further studies can be done in large settings.

## CONCLUSIONS

This study highlights that poor maternal knowledge regarding oral health is significant issue in eastern Nepal because most of pregnant women do not visit dentist for their dental problems. There is a gap in knowledge and practices related to oral and dental healthcare in women during pregnancy. Oral health not seen as priority, followed by costs was the common barriers to seek dental care during pregnancy. The study suggest the need for preventive strategies involving dentists and antenatal providers to improve maternal oral health in eastern Nepal by reinforcing need to maintain good oral hygiene during pregnancy and the importance of dental visits. Larger studies are required to get a proper national scenario regarding this issue.
